# Determination of plasma transcobalamin-II and zinc levels in newly–diagnosed and long–standing grand mal epileptic patients

**DOI:** 10.22088/cjim.14.2.199

**Published:** 2023

**Authors:** Amene Aman-Mohammady, Payam saadat, Durdi Qujeq, Karimollah Hajian-Tilaki, Kiarash Saleki

**Affiliations:** 1 Babol university of Medical sciences, Babol, Iran; 2 Mobility Impairment Research Center, Health Research Institute, Babol University of Medical Sciences, Babol, Iran; 3 Department of Biochemistry, Babol University of Medical Sciences, Babol, Iran; 4 Social Determinants of Health Research Center, Health Research Institute, Babol University of Medical Sciences, Babol, Iran; 5 Student Research Committee, Babol University of Medical Sciences, Babol, Iran; 6 USERN Office, Babol University of Medical Sciences, Babol, Iran; 7 Department of e-Learning, Virtual School of Medical Education and Management, Shahid Beheshti University of Medical Sciences(SBMU), Tehran, Iran.

**Keywords:** Transcobalamin-2, Zn, Epilepsy, Newly–diagnosed epilepsy, Grand mal seizure

## Abstract

**Background:**

The changes of plasma transcobalamin-II (TCII) and Zinc (Zn) Levels in epileptic patients are not clearly understood. The aim of the current study was to evaluate the plasma contents of TCII and Zn levels in newly–diagnosed epileptic seizure patients, long-standing grand mal epileptic patients following treatment with sodium valproate and healthy control group.

**Methods:**

Thirty patients aged 36.76±12.91 years with newly–diagnosed and thirty long-standing grand mal epileptic patients aged 35.56 ±12.77 years were diagnosed based on the clinical symptoms. The control subjects were picked out from healthy individuals and matched to the patients, aged 36.30 ±12.80 years. Plasma Zn and TCN-2 was evaluated via spectrophotometry at 546 nm and 450 nm, respectively, using chimerical kits.

**Results:**

Plasma level of TCII in the newly–diagnosed epileptic seizures patients and long-standing grand mal epileptic patients were significantly increased, compared to the healthy controls [14.89 ±3.24 and 21.84± 2.73 vs. 9.55±1.24, (n=30)], respectively. Plasma level of Zn was decreased in the newly–diagnosed epileptic seizure patients, while it was increased in long-standing grand mal epileptic patients compared to the control group [69.28± 6.41 and 80.56 ±6.12 and vs.75.80±1.59, (n=30)], respectively.

**Conclusion:**

This study suggests that sodium valproate may disrupt the homeostatic balance of TCII and Zn, and cause abnormality of their serum level in newly–diagnosed epileptic seizure patients and long-standing grand mal epileptic patients. Further research is recommended to identify the underpinning for these changes.

Zn is a cofactor for the glutamic acid decarboxylase enzyme. Defect in this biological catalyst can affect epileptic disorder (1)**. **Researchers showed that the untreated epilepsy patients presented unchanged Zn levels, while sodium valproate-treated epilepsy patients showed a meaningful increase in the contents of Zn level (2). However, other researchers reported no change in serum Zn level in some epileptic patients compared to the control group as described previously (3)**.** Previous study demonstrated that valproate is one of the most common drugs used as an anticonvulsive (4). Other study showed that similarity between some of the side effects that develop during long-term valproic acid (VPA) treatment and sign of Zn deficiency is high (5). Accumulating evidence has found that during VPA mono-therapy antioxidant status in blood of patients with epilepsy is changed. Researchers have evaluated some antioxidant enzymes were evaluated in patients with epilepsy and control subjects.

The antioxidant status of epileptic case was affected as a result of valproate monotherapy. Cases with uncontrolled epilepsy showed elevated Zn levels compared to seizure-free cases (6). It seems that Zn can play an antiepileptic role when given in appropriate doses (7). Growing evidence demonstrated that the epileptic patients with uncontrolled epilepsy have higher serum Zn compared to seizure-free patients (6). Antiepileptic drugs (AEDs) are important for the treatment of epilepsy (8, 9). Chemical materials are essential for regular function of the central nervous system (CNS), and researchers indicated that changes in element content of the body are effective on the incidence of epilepsy (10). Antiepileptic drugs can change the amount of trace elements in the body. Sodium valproate is a very effective anticonvulsant drug that is widely used in all types of epilepsy. Patients with epilepsy are at risk for Zn and selenium deficiency, both of which have antioxidant properties (7). Anticonvulsants such as sodium valproate produce high amounts of reactive oxygen species (ROS), which, unlike newer drugs such as topiramate and zonisamide, use Zn and selenium sources. Zn regulates the enzyme glutamic acid carboxylase, which plays a major role in the production of an inhibitory neurotransmitter called gamma amino butyric acid, which deficiency can lead to epileptic disorders. Zn in its bivalent cation form is abundant in the brain, in particular in the hippocampus. Recent evidence suggests that Zn is packaged within synaptic vesicles in the hippocampus and can be released along with other neurotransmitters (11).

Zn inhibits the activity of type A receptors for gamma amino butyric acid (GABA). The sensitivity of this receptor to Zn is due to its alpha subunit. Alpha 4, alpha 5, alpha 6 subunits are more sensitive than other alpha subunits. Alpha 4 and Alpha 5 are highly expressed in the hippocampus and possibly mediate any effect of Zn on GABAergic transducers in this region. Valproate may affect the intracellular distribution of Zn (12). Valproate induces Zn deficiency in experimental animals (13). Some studies in patients with epilepsy have shown that changes in the levels of some trace electrolytes, including Zn, affect the occurrence of seizures, and that reducing their sources in living organisms reduces the activity of enzymes that depend on them, resulting in cell destruction. TCII deficiency is an autosomal recessive disease marked by defective intestinal absorption of vitamin B12 (14). Epilepsy was observed in patients with TCII deficiency with extended duration of illness (15). 

Therefore, based on the evidence presented for the above factors and citing other studies, we aimed to evaluate the plasma levels of TC-II and Zn in adult epilepsy patients treated with sodium valproate as well as to determine the effects of their changes.

## Methods

This study was carried out in the Neurology Department, Rouhani Hospital, Faculty of Medicine, Babol University of Medical Sciences, Babol, Iran between November 2016 and January 2018. All subjects included in the project provided signed informed consent of the experimental protocol as recommended by the university ethics committee, and in agreement with the Helsinki Declaration. The Ethical Committee of Babol University approved the study (MUBABOL.REC.1395.179). 

Ninety subjects participated in the study: This case-control study included 30 patients with grand mal epilepsy (group III) treated with valproate sodium (15 mg/Kg/day, for 6 months), 30 patients with newly diagnosed epileptic seizures patients (group II), and 30 healthy controls (group I). All 60 patients were diagnosed with epilepsy based on the clinical symptoms and the observations of epileptic discharges in electroencephalography (EEG). The healthy subject group comprised 30 healthy individuals matched with the case group. The inclusion criteria were: I. No history of seizures (in group I patients); II. No history of grand mal epilepsy (in group II patients); III. No previous diagnosis of neurological disorders; IV. Not having used drug and Zn containing food in the past twelve months. V. Older than 20 years, an epilepsy duration of at least 6 months, and normal clinical examination. Exclusion criteria were: I. Subject had a disorder that affects the TCII and Zn content. II. Subjects suffer from diseases such as diabetes, anemia, kidney and cardiovascular disease.

**Target population and sampling: **After a 12 hour night fasting, 3 ml venous blood samples were collected from the subjects. Then, the plasma Zn was measured by spectrophotometry using a chimerical kit. A highly sensitive procedure for spectrophotometric determination of Zn has been developed. At pH 4.6, in ethanol-water medium and in the presence of di-2-pyridyl ketone salicyloylhydrazone, Zn forms a yellow complex which has maximum absorption at 375 nm. The detection limit of this method was 69.28± 6.41 for Zn. Plasma TCII was measured by spectrophotometry at 450 nm using a chimerical kit. Using this technique, levels of TCII were determined in 90 subjects. The detection limit of this method was 14.89 ±3.24.

**Statistics: **One–way analysis of variance (ANOVA) was used for statistical analysis. Comparison was made by students t-test, P<0.05 was considered to be statistical significant. Statistical packages at 80% power and 95% confidence interval then estimated 30 participants should be included in each group.

## Results

**Figure 1:**
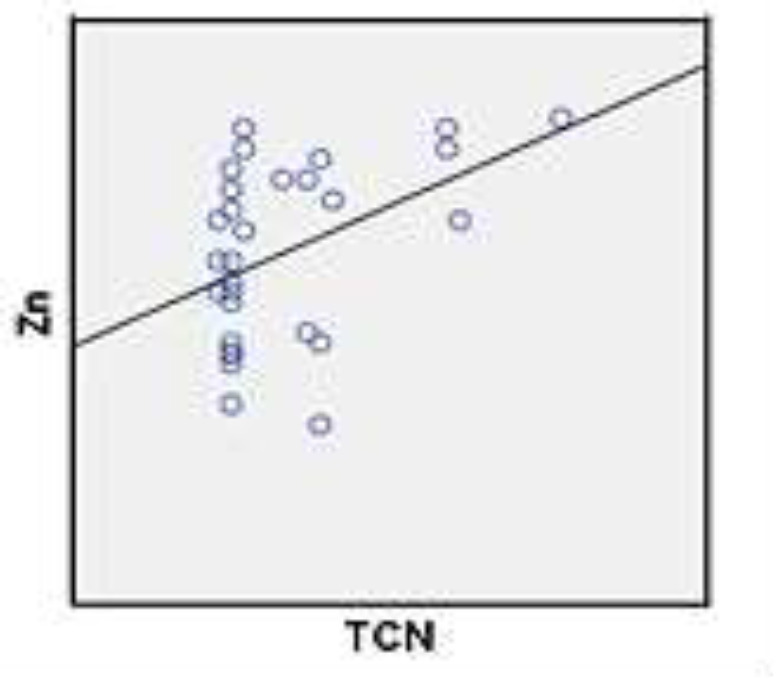
The correlation of mean value of Zn and Transcobalamin plasma levels in healthy control group

**Figure 2:**
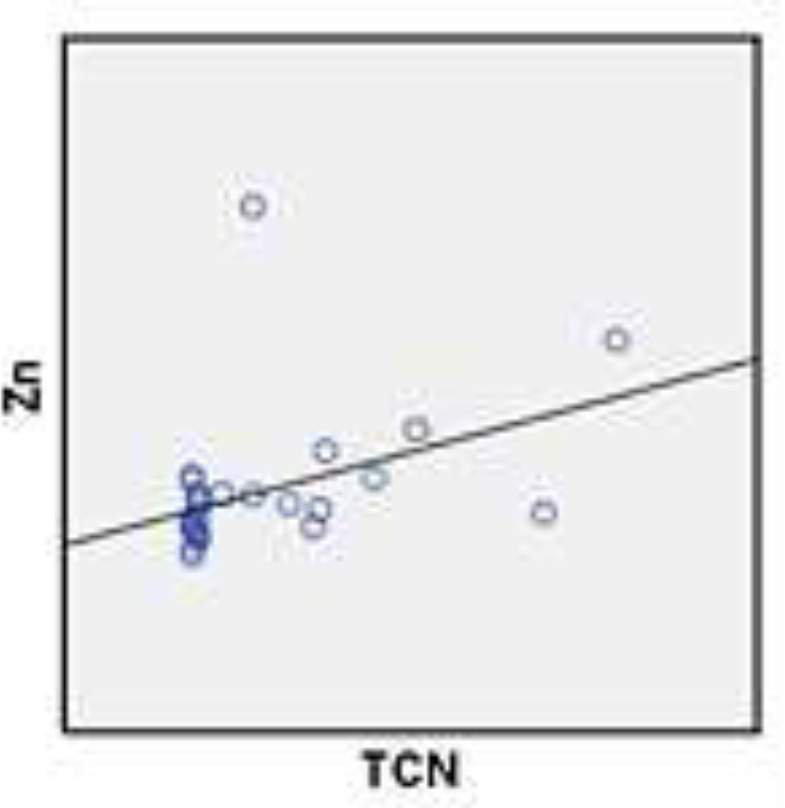
The correlation of mean value of Zn and Transcobalamin plasma levels in standing grandmal epileptic patients treatment with sodium valproate

**Figure 3:**
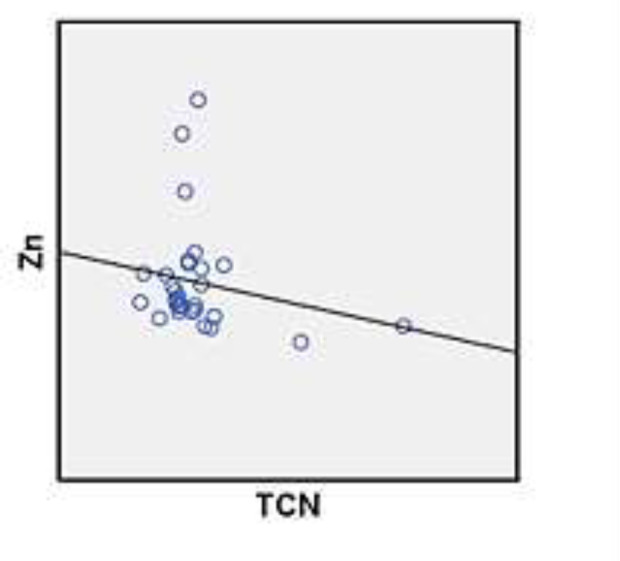
The correlation of mean value of Zn and Transcobalamin plasma levels in newly-diagnosed patients

As shown in table 1 the plasma levels of TCII level in long–standing grand mal epileptic patients were significantly higher following treatment compared to those newly–diagnosed (P=0.04) and healthy controls. Also, the plasma Zn levels of long–standing grand mal epileptic patients following treatment was significantly higher than newly–diagnosed epileptic seizures patients (P=0.31) and controls (Table 1). The correlation between mean plasma levels of TCII and Zn of healthy controls, newly–diagnosed patients and long–standing grand mal epileptic patients’ treatment with sodium valproate was demonstrated in Table 2. The correlation of mean value of ZN and TCII plasma levels in healthy control group was directly positive (figure 1)**.**The correlation of mean value of Zn and TCII plasma levels in long**-**standing grand mal epileptic patients treated with sodium valproate was positive (figure 2). The correlation of mean value of Zn and TCII plasma levels in newly–diagnosed patients was negative (figure 3).

**Table 1:** Comparison between mean plasma levels of TC-II and Zn of healthy controls, newly–diagnosed patients and long–standing grand mal epileptic patients treated with sodium valproate

**Variable C V W**
** N=30 N=30 N=30 p-value**
Transcobalamin(ng/ml) 9.55 ± 1.24 21.84 ±2.73 14.89 ± 3.24 0.04
Zn(µg/dl) 75.80 ± 1.59 80.56 ± 6.12 69.28 ± 6.41 0.31

C: Control (group I); V: newly–diagnosed patients (group II); W:long –standing grand mal epileptic patients (Group III).Data presented as Mean±SD

**Table2:** Correlation between mean plasma levels of transcobalamin and Zn of healthy controls, newly–diagnosed patients and long –standing grand mal epileptic patients treatment with sodium valproate

		Transcobalamin (ng/ml)		Zn (µg/dl)	
		r	p	r	p
C	Transcobalamin Zn	- 0.43	- 0/01	0.43 -	0.01 -
V	Transcobalamin Zn	- -0.18	- 0.32	-0.18 -	0.32 -
W	Transcobalamin Zn	0.41 -	0.02 -	- 0.41	- 0.02

C: Control (group I); V: newly –diagnosed patients (group II); W: long–standing grand mal epileptic patients (group III). Data presented as Mean±SD.

## Discussion

Epilepsy is a disorder categorized by recurrent seizures and leads to changes in neuronal death and neurogenesis. Recently, the search for new biochemical factors has focused on epilepsy. It is widely accepted that biochemical factors are implicated in various metabolic processes such as epilepsy. Currents experiments from our laboratory indicate that plasma level of TC-II level are involved in the newly–diagnosed epileptic seizures patients and long –standing grand mal epileptic patients.

 Plasma level of TCII level in the newly–diagnosed epileptic seizures patients and long–standing grand mal epileptic patients were increased compared to that in the healthy controls, but difference were not significant. Also, plasma level of Zn in long –standing grand mal epileptic patients was increased, while it was decreased in the newly–diagnosed epileptic patients compared to the healthy controls, but difference were not significant.

Under sodium valproate treatment, serum Zn level in long–standing grand mal epileptic patients was higher compared to the control group. Our result is in agreement with a previous study that found increased serum Zn in epileptic patients (16). In a study by Saad et al., Zn levels did not change in untreated epileptic patients, but in patients treated with sodium valproate, plasma Zn levels were significantly higher. The true mechanism for the possible effect of antiepileptic drugs on Zn concentrations is not well-known. However, it can be suggested that sodium valproate binds to the element Zn and protects GABA from the inhibitory effect of Zn, thereby increasing the concentration of GABA (16). In a study by Kumar et al, however, febrile seizure patients showed lower Zn levels. No meaningful variation in serum Zn was found in association with outcome and consciousness level in any of the experiment subgroups (17). In a study by Sarangio et al., a statistically significant increase in Zn concentration was observed in epileptic patients who used antiepileptic drugs as monotherapy. It seems that this increase could be due to increased levels of antioxidant enzymes and Zn is an integral part of them (18). But, our result is not in agreement with previous studies that found a significantly decreased serum Zn in epileptic patients (19, 11). 

In a study by Saboktakin et al., plasma Zn levels were significantly lower in epileptic patients treated with antiepileptic drugs (11). In a study by Motta et al., a decrease in plasma copper levels was observed in epileptic patients who used antiepileptic drugs for a long time but had no effect on plasma Zn levels (19). This controversy of results may be due to change of antioxidant enzymes levels and neurotransmitter content related to Zn metabolism. The true mechanism of the possible effect of sodium valproate on Zn metabolism is not clearly understood. The limitation of this study was small sample size. We present an interesting analysis of the serum Zn and TCII levels in individuals with the newly–diagnosed epileptic patients and long–standing grand mal epileptic patients. Our study is interesting because it underscores the concern about biochemical factors. Our findings indicate that plasma Zn and TCII levels in long–standing grand mal epileptic patients under sodium valproate treatment are higher compared to the newly–diagnosed epileptic patients and healthy controls. Thus, molecular analysis should be done in order to establish a firm diagnosis in subjects with grand mal epileptic patients. We suggest that our findings will help the development of future extensive investigations into the therapy and mechanism of epilepsy in order to enhance the life quality of epileptic patients.
